# Serum uric acid as a risk factor for rejection after deceased donor kidney transplantation: A mono-institutional analysis of paired kidneys

**DOI:** 10.3389/fimmu.2022.973425

**Published:** 2022-12-12

**Authors:** Fuxun Zhang, Jiayu Liang, Yang Xiong, Fan Zhang, Kan Wu, Wei Wang, Jiuhong Yuan, Tao Lin, Xianding Wang

**Affiliations:** Department of Urology, Institute of Urology, West China Hospital, Sichuan University, Chengdu, Sichuan, China

**Keywords:** kidney transplantation, donor, recipient, rejection, risk factor

## Abstract

**Background:**

Deceased donor kidney transplantation (DDKT) is a major therapeutic option for patients with end-stage renal diseases. Although medical techniques improved in recent years, acute or chronic rejection after DDKT is not uncommon and often results in poor graft survival. Therefore, the determination of risk factors is very important to stratify patients and to improve outcomes. This study aims to evaluate the risk factors for treated rejection (TR) of patients after DDKT.

**Methods:**

Clinical data of deceased donors and corresponding recipients were retrospectively collected. The primary outcome was TR defined as the treatment for rejection within 24 months after DDKT. Univariate comparisons of baseline characteristics were performed with Chi-square test, t-test, and Mann–Whitney U test. Logistic regression was constructed to analyze potential risk factors. Receiver operating characteristic (ROC) curve and Jordan index were generated to determine the optimal cutoff value. The association between continuous variables and TR was examined and visualized by using restricted cubic spline (RCS) models.

**Results:**

Data of 123 deceased donors and 246 recipients were obtained and analyzed. The median age was 41 (4–62) years for recipients and 39 (1–65) years for donors. The recipients who died or suffered graft loss during the follow-up period were 8 (3.3%) and 12 (4.9%), respectively. After univariate analysis and subsequent multivariate analysis, the preoperative serum uric acid (OR, 2.242; 95% CI, 1.037–4.844; *P* = 0.040), platelet (OR, 2.163; 95% CI, 1.073–4.361, *P* = 0.031), absolute neutrophil count (OR, 2.183; 95% CI, 1.025–4.649; *P* = 0.043), and HLA-DQ mismatch (OR, 2.102; 95% CI, 1.093–4.043; *P* = 0.026) showed statistical significance. RCS models showed that patients with higher levels of uric acid had increased risk of TR.

**Conclusions:**

Serum uric acid and other three indicators were found to be the independent risk factors for TR, which may contribute to stratify patients and develop personalized regimen in perioperative period.

## Introduction

Worldwide, kidney transplantation from deceased donors is a major therapeutic option for patients with end-stage kidney diseases (1). With the improvement of medical techniques in recent years, long-term survival of recipients after deceased donor kidney transplantation (DDKT) appears to be possible. Thus, studies on complications related to graft failure and mortality in organ transplantation are essential (2). Although several risk factors for DDKT outcomes have been documented before, current evidence reveals that short-term adverse events including acute rejection and delayed graft function (DGF) are not rare, leading to increased incidence of graft loss, re-transplantation, and even death (3, 4).

On the other hand, it is reported that kidneys from deceased donors may suffer from various injurious factors during the donation (5). Among them, the source of donor kidney has been suggested as an important factor related to different outcomes of transplantation (5). Kidneys from the donation after cardiac death (DCD) donors may be susceptible to injury by warm ischemia and subsequently experience a higher incidence of DGF, and kidneys from the donation after brain-stem death (DBD) could incur greater metabolic disturbance and inflammatory response (6, 7). However, limited data and inconsistent opinions on the risk factors of graft survival in DDKT still exist (8, 9). Therefore, elements on graft survival require further investigation.

Indeed, the association of rejection in DBD with inferior graft outcomes has been established previously (6). Meanwhile, the definition, diagnosis, and treatment of rejection have been refined (10). However, the outcomes of DDKT can be affected by complicated factors, including specific allocation, healthcare, and surgical procedures, producing inevitable confound factors and statistical bias. Thus, a comprehensive assessment of risk factors based on paired donors and recipients might be conducive to reduce bias and make correct allocation and adequate preparation for transplants. Taken together, the determination of risk factors is important to stratify patients and improve graft survival (1). This study aims to evaluate the risk factors for treated rejection (TR) after DDKT basing on the data of paired kidneys.

## Methods

### Patients and data collection

We retrospectively collected the data of both donors and recipients performed DDKT between 2015 and 2018 in West China Hospital. The end of follow-up period in this study is December 2020. After the exclusion of eight donors and 16 paired recipients due to lack of major preoperative information, insufficient follow-up data, dual organ transplantation, and donor age older than 65, we included 123 donors and 246 recipients and analyzed their data (**Supplementary Figure 1**). Baseline characteristics included transplantation-related features and recipient and donor characteristics (**Tables 1**–**3**). Recipient age was not restricted, and donor–recipient characteristics were matched correspondingly to analyze covariates and control confounding variables better. For each recipient, clinical and laboratory information within 2 years after transplantation was obtained.

**Table 1:** Baseline characteristics of recipients and their association with treated rejection.

Variables (*n* = 246)	Treated rejection		
	Yes	No	*P*-value
Gender, *n* (%)			0.488
Male	33 (13.4)	146 (59.3)	
Female	15 (6.1)	52 (21.1)	
Blood type, *n* (%)			0.235
O	20 (8.1)	61 (24.8)	
A	14 (5.7)	56 (22.8)	
B	7 (2.8)	58 (23.6)	
AB	7 (2.8)	23 (9.3)	
Primary renal disorders, *n* (%)			0.467
Unknown	36 (14.6)	158 (64.2)	
Glomerulonephritis-related	12 (4.9)	40 (16.3)	
Diabetes mellitus, *n* (%)			0.613
No	46 (18.7)	186 (75.6)	
Yes	2 (0.8)	12 (4.9)	
DGF, *n* (%)			0.284
No	31 (12.7)	142 (58.2)	
Yes	17 (7.0)	54 (22.1)	
Preoperative dialysis, *n* (%)			0.911
HD	38 (16.0)	149 (62.6)	
PD/PD combined with HD	10 (4.2)	41 (17.2)	
Previous kidney transplantation			**0.096**
No	48 (19.5)	187 (76.0)	
Yes	0 (0.0)	11 (4.5)	
Postoperative infections, *n* (%)			0.558
None	26 (10.6)	125 (50.8)	
Infection			
Pulmonary	19 (7.7)	46 (18.7)	
Urinary	3 (1.2)	7 (2.8)	
Others	0 (0.0)	20 (8.1)	
Death, *n* (%)			0.158
Survival	48 (19.5)	190 (77.2)	
Died	0 (0.0)	8 (3.3)	
Overall graft loss, *n* (%)			0.318
No	47 (19.1)	187 (76.0)	
Yes	1 (0.4)	11 (4.5)	
**Variables**	***n* = 246**		***P*-value**
Age, years (range)	41 (4–62)		0.687
BMI, kg/m^2^ (range)	21.5 (13.7–38.2)		**0.003**
Duration of preoperative dialysis, months (range)	24 (1–240)		0.540
Time of rejection occurrence after DDKT	14 (1–600)		0.886
Preoperative laboratory workup			
Serum creatinine, μmoI/L (range)	868 (254–1722)		0.119
Serum CysC, mg/L (mean ± SD)	7.7 ± 2.2		0.784
eGFR, ml/min (mean ± SD)	8.5 ± 3.9		0.368
FBG, mmoI/L (range)	5.25 (3.44 - 27.75)		0.494
UA, μmoI/L (mean ± SD)	387.5 ± 116.8		**0.004**
Hb, g/dl (mean ± SD)	113.6 ± 21.6		0.173
PLT, 10^9^ (range)	164 (60–401)		**0.034**
WBC, 10^9^ (range)	6.61 (2.90–13.45)		**0.003**
ALC, 10^9^ (range)	1.15 (0.11–2.77)		0.544
AMC, 10^9^ (range)	0.33 (0.07–1.02)		0.360
ANC, 10^9^ (range)	4.65 (1.76–10.49)		**0.003**
NLR, ratio (range)	4.12 (1.29–67.91)		0.900
HDL, mmoI/L (range)	1.25 (0.10–5.14)		0.313
LDL, mmoI/L (range)	2.12 (0.36–7.94)		0.774
Triglyceride, mmoI/L (range)	1.37 (0.01–108.00)		0.773

BMI, body mass index; DDKT, deceased donor kidney transplantation; HD, hemodialysis; PD, peritoneal dialysis; DGF, delayed graft function; CysC, cystatin C; eGFR, estimated glomerular filtration rate; FBG, fasting blood-glucose; UA, uric acid; Hb, hemoglobin; PLT, platelet; WBC, white blood cell; ALC, absolute lymphocyte count; ANC, absolute neutrophil count; AMC, absolute monocyte count; NLR, neutrophil-to-lymphocyte ratio; HDL, high-density lipoprotein; LDL, low-density lipoprotein. Bold figures indicate as statistical significance at P < 0.10.

**Table 2:** Transplantation-related features and their association with treated rejection.

Variables (*n* = 246)	Treated rejection		
	Yes	No	*P*-value
CIT			0.855
PRA I, *n* (%) (Luminex technology)			0.211
PRA ≤ 30%	48 (19.8)	190 (78.2)	
PRA > 30%	0 (0.0)	5 (2.1)	
PRA II, *n* (%)			0.576
PRA ≤ 30%	47 (19.3)	193 (79.1)	
PRA > 30%	1 (0.4)	3 (1.2)	
HLA mismatch, *n* (%)			
A			0.883
0	6 (2.5)	31 (12.9)	
1	30 (12.5)	121 (50.4)	
2	11 (4.6)	41 (17.1)	
B			**0.023**
0	0 (0.0)	3 (1.3)	
1	11 (4.6)	77 (32.1)	
2	36 (15.0)	113 (47.1)	
DR			0.247
0	2 (0.8)	6 (2.5)	
1	17 (7.1)	101 (42.1)	
2	28 (11.7)	86 (35.8)	
DQ			**0.028**
0	0 (0.0)	13 (6.0)	
1	22 (10.1)	103 (47.5)	
2	20 (9.2)	59 (27.2)	
Type I			0.202
0	1 (0.4)	7 (2.8)	
1	0 (0.0)	15 (6.1)	
2	13 (5.3)	67 (27.2)	
3	27 (11.0)	79 (32.1)	
4	7 (2.8)	30 (12.2)	
Type II			**0.067**
0	1 (0.4)	9 (3.7)	
1	4 (1.6)	22 (8.9)	
2	17 (6.9)	83 (33.7)	
3	7 (2.8)	30 (12.2)	
4	19 (7.7)	54 (22.0)	
Induction, *n* (%)			0.722
ATG	21 (8.6)	88 (35.9)	
BSX/others	27 (11.0)	109 (44.5)	

CIT, cold ischemic time; PRA, panel-reactive antibody; HLA, human leukocyte antigen; ATG, anti-thymocyte globulin; BSX, basiliximab.

Bold figures indicate as statistical significance at P < 0.10.

**Table 3:** Baseline characteristics of donors and their association with treated rejection.

Variables	*n* = 246	*P*-value
Age, years (range)	39 (1–65)	0.808
Gender, *n* (%)		0.535
Male	89 (72.4)	
Female	34 (27.6)	
Blood type, *n* (%)		0.505
O	46 (37.4)	
A	32 (26.0)	
B	34 (27.6)	
AB	10 (8.1)	
Diabetes mellitus, *n* (%)		0.494
No	115 (93.5)	
Yes	1 (0.8)	
Hypertension, *n* (%)		0.545
No	96 (78.0)	
Yes	25 (20.3)	
Viral hepatitis, *n* (%)		0.853
HBV positive	7 (5.7)	

HBV, hepatitis B virus. Bold figures indicate as statistical significance at P < 0.10.

### Clinical outcome and definitions

The primary clinical outcome was TR defined as the treatment for rejection within 24 months after the DDKT. DGF was defined as receiving dialysis within 1 week after the transplantation. Overall graft loss means regaining permanent dialysis after the transplantation or death with functional graft by any cause. Cold ischemic time (CIT) was established by the time from cold storage to reperfusion following implantation. Serum creatinine of recipients at implantation and within 24 months after transplantation was available for evaluating renal function and graft performance. Human leukocyte antigen (HLA) mismatch according to the UK allocation policy was defined as the incongruity of HLA-A, HLA-B, HLA-DR, HLA-DQ, HLA-Type I, and HLA-Type II between donor and recipient. Baseline characteristics of the included 123 pairs and transplantation were assessed as covariant for exploring potential risk factors.

### Statistical analysis

Statistical analyses in this study were performed using SPSS 23.0 (SPSS, Inc., Chicago, USA). Mean value and standard deviation or median with ranges were calculated for continuous variables. Rates or proportions were calculated for categorical variables. The Kolmogorov–Smirnov test was used to indicate the distribution of variables. Univariate comparisons between transplants with rejection versus without rejection were made with Chi-square tests, t-test, and Mann–Whitney U test when appropriate. Meanwhile, variables were deemed to be statistically significant at *P*-values less than 0.10 in the correlation analyses, which might be conducive to seek potential correlation and risk factors. Logistic regression model was constructed to analyze potential risk factors. Significant variables in correlation analyses were incorporated into the regression model. In addition, estimated glomerular filtration rate (eGFR) was included in multivariate analysis for adjusting the effect of renal function on outcomes.

In multivariate analysis, the stepwise regression method was chosen to prevent multicollinearity among variables. Receiver operating characteristic (ROC) curve and corresponding Jordan index (subtract 1 from the sum of sensitivity and specificity) were generated to determine the optimal cutoff value for continuous variables in the regression model. The area under the curve (AUC) in ROC analysis is considered as a predictor to distinguish patients with or without TR. The AUC between 0.8 and 0.9 represents a high-rank results test, and the AUC between 0.5 and 0.6 represents a poor test. The peak point locating at where the sensitivity and specificity were maximized simultaneously was highest cutoff point. The association of variables on continuous scales with primary outcome was examined through the use of restricted cubic spline (RCS) regression models with multifactorial adjustment. RCSs with equally spaced percentiles (25th, 50th, 75th, and 95th) were used to capture the association, to visualize the relationship, and to test the potential non-linearity. In regression analysis, *P*-values were two-sided, and the statistical significance was defined as P < 0.05.

## Results

### Characteristics of study population and their relevance with TR

Of 246 DDKT recipients, 128 (52.0%) were from donors suffered severe craniocerebral injury, 84 (34.2%) from intracranial hemorrhage, and 34 (13.8%) from other diseases such as intracranial tumors. The median (range) age was 41 (4–62) years for recipients and 39 (1–65) years for donors. The recipients who died or suffered graft loss during the follow-up period were 8 (3.3%) and 12 (4.9%), respectively. Maintenance immunosuppressive therapy of recipients was the combination of tacrolimus, mycophenolate mofetil (MMF), and prednisone (97%) or the combination of cyclosporin, MMF, and prednisone (3%). Recipients who suffered from cardiovascular events, hyperparathyroidism, diabetes, anemia, and psychiatric disorders following transplantation were 6 (2.4%), 5 (2.0%), 5 (2.0%), 2 (0.8%), and 3 (1.2%), respectively. Of 246 recipients, 48 (19.5%) were diagnosed as TR. Overall, 26 (10.6%) were antibody-mediated rejection, 18 (7.3%) were T-cell–mediated rejection, and 4 (1.6%) were mixed form. Their levels of serum creatinine and cystatin C seem to be higher compaed with non-TR at same time within 24 months, indicating the adverse effect of TR on renal function (**Supplementary Figure 2**).

The median (range) body mass index (BMI) of recipients was 21.5 (13.7–38.2) kg/m^2^, which showed potential correlation with TR (*P* = 0.003). Of transplantation-related variables, HLA-B mismatch, HLA-DQ mismatch, and HLA-Type II showed significant correlation with TR (*P* = 0.023, 0.028, and 0.067, respectively). Unexpectedly, several parameters of preoperative laboratory workup showed robust association with TR. The uric acid (UA) (*P* = 0.004), platelet (PLT) (*P* = 0.034), white blood cell (WBC) (*P* = 0.003), and absolute neutrophil count (ANC) (*P* = 0.003) showed statistical significance of association with TR. All characteristics of transplants and their relevance to TR are shown in **Tables 1**–**3**.

### Variables and risk fact of TR

**Figure 1:**
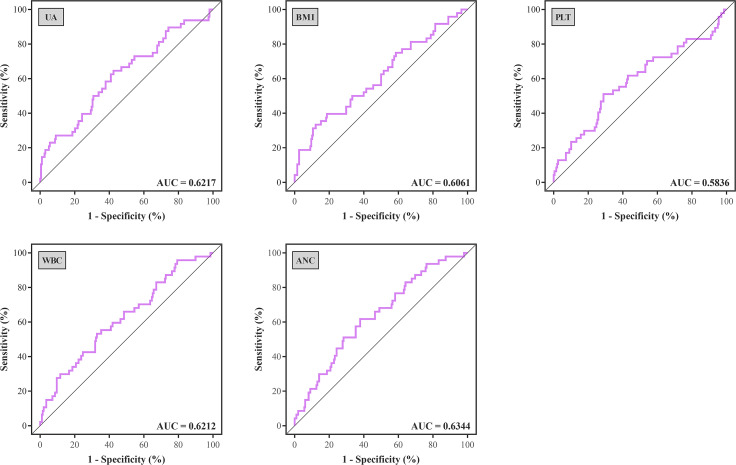
ROC curves were generated to determine the optimal cutoff value of UA, BMI, PLT, WBC, and ANC for treated rejection. ROC, receiver operating characteristic; AUC, area under the curve; UA, uric acid; BMI, body mass index; PLT, platelet; WBC, white blood cell; ANC, absolute neutrophil count.

Optimal cutoff values of meaningful continuous variables in preliminary correlations analyses were confirmed *via* ROC curve and corresponding Jordan index (**Figure 1**). After that, dichotomous variables were generated and entered into univariate and multivariate analysis to explore independent risk factors. In univariate analysis, several variables of recipients and transplantation were strongly associated with TR (**Table 4**). Among them, BMI (OR, 3.145; 95% CI, 1.500–6.596; *P* = 0.002), preoperative UA (OR, 2.309; 95% CI, 1.207–4.419; *P* = 0.011), PLT (OR, 2.519; 95% CI, 1.317–4.818; *P* = 0.005), WBC (OR, 2.273; 95% CI, 1.193–4.330; *P* = 0.013), and absolute lymphocyte count (ALC) (OR, 2.532; 95% CI, 1.317–4.868; *P* = 0.005) of recipients demonstrated the potential risk for TR. Meanwhile, HLA-B mismatch (OR, 2.325; 95% CI, 1.134–4.764; *P* = 0.021) and HLA-DQ mismatch (OR, 1.950; 95% CI, 1.057–3.600; *P* = 0.033) might be risk factors for TR. Previous kidney transplantation did not acquire statistical assignment, perhaps due to the small sample (*n* = 11).

**Table 4:** Risk factors for treated rejection in univariate and multivariate analysis.

Risk factors	Univariate analysis		Multivariate analysis	
	OR (95% CI)	*P*-value	OR (95% CI)	*P*-value
Recipient-preoperative				
BMI > 24.5 kg/m^2^	3.145 (1.500–6.596)	**0.002**	2.278 (0.965–5.377)	0.060
UA > 400 μmoI/L	2.309 (1.207–4.419)	**0.011**	2.242 (1.037–4.844)	**0.040**
PLT > 185 × 10^9^	2.519 (1.317–4.818)	**0.005**	2.163 (1.073–4.361)	**0.031**
WBC > 7.3 × 10^9^	2.273 (1.193–4.330)	**0.013**	1.706 (0.799–3.644)	0.168
ANC > 5.0 × 10^9^	2.532 (1.317–4.868)	**0.005**	2.183 (1.025–4.649)	**0.043**
eGFR	0.948 (0.846–1.603)	0.360	0.989 (0.892–1.096)	0.830
Transplantation-related				
HLA mismatch				
B	2.325 (1.134–4.764)	**0.021**	1.836 (0.875–3.853)	0.108
DQ	1.950 (1.057–3.600)	**0.033**	2.102 (1.093–4.043)	**0.026**
Type II	1.286 (0.966–1.713)	0.085	0.998 (0.401–2.484)	0.997

BMI body, mass index; UA, uric acid; PLT, platelet; WBC, white blood cell; ANC, absolute neutrophil count; eGFR, estimated glomerular filtration rate; HLA, human leukocyte antigen; OR, odds ratio. Bold figures indicate as statistical significance at P < 0.05.

**Figure 2:**
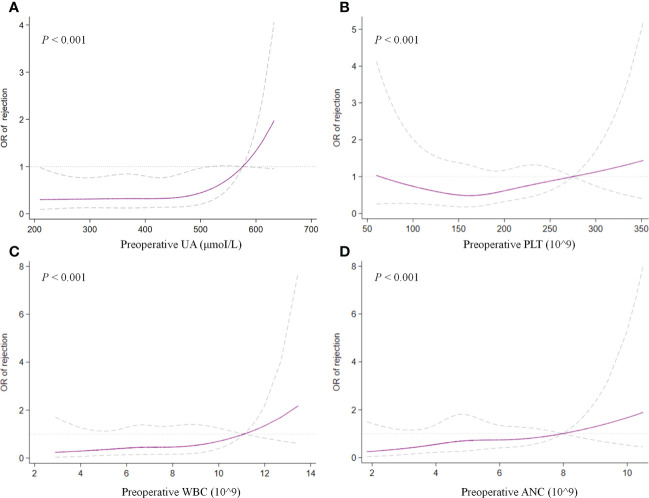
Restricted cubic splines examined the association of preoperative UA **(A)**, PLT **(B)**, WBC **(C)**, and ANC **(D)** with treated rejection. UA, uric acid; PLT, platelet; WBC, white blood cell; ANC, absolute neutrophil count.

In the multivariate analysis, UA > 400 μmoI/L (OR, 2.242; 95% CI, 1.037– 4.844; *P* = 0.040), platelet > 185 × 10^9^ (OR, 2.163; 95% CI, 1.073–4.361; *P* = 0.031), ANC > 5.0 × 10^9^ (OR, 2.183; 95% CI, 1.025–4.649; *P* = 0.043), and HLA-DQ mismatch (OR, 2.102; 95% CI, 1.093–4.043; *P* = 0.026) still showed statistical relevance and could be considered as the independent predictors for TR (**Table 4**). On the other hand, after multifactorial adjustment for covariates, RCS regression models indicated that patients with higher levels of preoperative serum UA had increased risk of rejection with some non-linearity (*P* < 0.001) (**Figure 2A**). The count of PLT, WBC, and ANC showed similar relationships with rejection (*P* < 0.001) (**Figures 2B–D**). Of note, RCSs examined the association of WBC and ANC with rejection on a more linear scale (**Figures 2C, D**).

## Discussion

We conducted the comprehensive analysis of 123 donors and 246 recipients and detected several possible risk factors for TR. In this study, donors’ profiles were matched with corresponding recipients for reducing potential selection biases in the evaluation of risk factors. Given the great infectious risk from over-immunosuppression caused by imbalance between immunosuppressive protocols and immunological rejection, appropriate stratification of recipients is important (11). Not only could the induction regimen individually tailored for each recipient, but also immunosuppression medication be personalized in line with immunological hazard. Hence, the assessment of risk factors for rejection would be beneficial to improve graft survival and long-term prognosis of patients.

Re-transplantation, grafts from deceased donors, and high level of panel-reactive antibody (PRA) have been reportedly associated with increased risk of graft loss and rejection after transplantation (12, 13). In the current cohort, these risk factors were also evaluated, and none of them demonstrated significant relevance to rejection in multivariate analysis, which might be attributed to the short-term follow-up and small population size that is not enough to prove these immunological associations. Graft matching remains a key element in allocation algorithm and selection of immunosuppressive agents, and poor HLA matching is associated with unwanted transplant outcomes (14). However, apart from HLA-DQ mismatch as an independent predictor of rejection that was confirmed (OR, 2.197; 95% CI, 1.119–4.317; *P* = 0.022), we also noted that several unexpected indicators from hemato-biochemical work-up of recipients showed statistical significance in the regression model. Precisely, UA with cutoff value of 400 μmoI/L, PLT with 185 × 10^9^, and ANC with 5.0 × 10^9^ exhibited robust association with TR and maybe considered as the risk factors for it.

Platelet and neutrophil have been deemed as the surrogates for inflammatory severity that is positively correlated prognosis in several diseases (15, 16). It is estimated that these indicators or ratios could reflect the systemic inflammation that might have adverse effects on hematologic cell lines and subsequently result in alteration of their ratios (16, 17). Current study demonstrated an independent positive correlation of both PLT and ANC with TR (OR, 2.202; 95% CI, 1.051–4.617; *P* = 0.037; and OR, 2.164; 95% CI, 1.018–4.599; *P* = 0.045, respectively) (**Figures 2B, D**). Our hypothesis is that elevated preoperative PLT and ANC of recipients maybe represent robust inflammatory response or over-activated immune system by any cause, which may drive the pathogenesis of rejection.

Another unexpected finding of our analysis was that preoperative serum UA levels revealed an independent association with TR in multivariate analysis (OR, 2.132; 95% CI, 1.016–4.476; *P* = 0.045). To test potential non-linearity, RCS regression models were used, which showed that the risk of rejection was relatively flat until UA was around 450 μmoI/L and then began to increase rapidly afterward, supporting the association between UA and TR. However, the comprehensive effect of UA on graft outcomes still remains controversial in published studies (18). Previous studies with complete and lengthy follow-up found an association between UA and outcomes of kidney transplantation (19, 20). Lower or normal levels of serum UA after transplantation might be an independent predictor of better graft outcomes in the long run (20). Recently, hyperuricemia was found to be related to increased death-censored graft loss but not an independent factor for long-term allograft function (21). Similarly, this study found no obvious correlation between preoperative serum UA and long-term renal function of allograft (**Supplementary Figure 3**).

It is unknown why UA could be an independent risk factor for TR in our study. Although hyperuricemia could result in deterioration of renal disease by inducing endothelial dysfunction and inflammatory dysregulation, it is hard to identify that UA is an immediate cause of renal disease due to unclear causal link between elevated UA and impaired renal function (22). Nevertheless, to our knowledge, the association of decreased UA with reduced graft-versus-host disease (GVHD) in allogeneic stem cell transplantation (allo-SCT) has been verified by animal models, and the level of serum UA could be used as a predictor for allo-SCT outcome (23). We therefore assume that the higher level of UA might initiate non-infectious inflammation and contribute to immune reconstitution, which increases the risk of rejection. Meanwhile, it is not unusual that hyperuricemia could be concomitant with end-stage renal diseases. Thus, although further studies are needed to confirm our results, it is necessary to address the hyperuricemia and decrease the UA during perioperative period.

In addition to retrospective design, this study has several inherent limitations. On the one hand, although the characteristics of donors were matched with recipients to control potential confounding factors, regression residual is a significant and iterative element in observational studies. On the other hand, for minimizing the negative effects of multicollinearity, the stepwise method was adopted in the regression model, which might marginalize some variables due to those with more statistical weight. Finally, our data from a single center may limit the potentially clinical application in other settings. However, although this retrospective study based on a single-center cohort and the results need further validation, the heterogeneity of large dataset from multicenter or even transnational registry could be significantly reduced in the current analysis.

## Conclusions

Our study revealed that several preoperative parameters might be the independent risk factors for TR after DDKT. In contrast to the previous studies, we found that serum UA may represent an independent risk factor for TR. Although further validation is required, decreasing preoperative UA of recipients might be beneficial to reduce rejection and improve graft survival in DDKT. Moreover, the determination of these inexpensive and potentially modifiable indicators may contribute to stratify patients and develop personalized regimen in perioperative period.

## Data availability statement

The raw data supporting the conclusions of this article will be made available by the authors, without undue reservation.

## Ethics statement

Written informed consent was obtained from the individual(s) for the publication of any potentially identifiable images or data included in this article. This study was approved by the Institutional Ethical Committee of the West China Hospital.

## Author contributions

FXZ: project development, data collection, data analysis, and manuscript writing. JL: project development, data analysis, and data collection. YX: data analysis. FZ: data analysis. KW: data collection. WW: data collection. JY: project development and data analysis. TL and XW: project development, data analysis, manuscript correction, and manuscript editing. All named authors meet the ICMJE criteria for authorship in this article, take responsibility for the integrity of the work as a whole, and have given their approval for this version to be published.

## Supplementary material

Supplementary Figure 1 Flow chart.

Supplementary Figure 2 Changes of serum creatinine (A) and Cys C (B) in patients grouped by rejection. * means significant difference of cross-sectional data. Data are means ± SD.

Supplementary Figure 3 Changes of serum creatinine (A) and Cys C (B) in patients grouped by preoperative serum uric acid (400 μmoI/L). * means significant difference of cross-sectional data. Data are means ± SD.

## References

[1] Summers DM Johnson RJ Allen J Fuggle SV Collett D Watson CJ . Analysis of factors that aff ect outcome after transplantation of kidneys donated after cardiac death in the UK: a cohort study. Lancet (2010) 376(9749):1303–11. doi: 10.1016/S0140-6736(10)60827-6 20727576

[2] Morrissey PE Monaco AP . Donation after circulatory death: current practices, ongoing challenges, and potential improvements. Transplantation (2014) 97(3):258–64. doi: 10.1097/01.TP.0000437178.48174.db 24492420

[3] Troppmann C Gillingham KJ Benedetti E Almond PS Gruessner RW Najarian JS . Delayed graft function, acute rejection, and outcome after cadaver renal transplantation. the multivariate analysis. Transplantation (1995) 59(7):962–8. doi: 10.1097/00007890-199504150-00007 7709456

[4] Zens TJ Danobeitia JS Leverson G Chlebeck PJ Zitur LJ Redfield RR . The impact of kidney donor profile index on delayed graft function and transplant outcomes: A single-center analysis. Clin Transplant (2018) 32(3):e13190. doi: 10.1111/ctr.13190 29314286 PMC6455919

[5] Summers DM Watson CJ Pettigrew GJ Johnson RJ Collett D Neuberger JM . Kidney donation after circulatory death (DCD): state of the art. Kidney Int (2015) 88(2):241–9. doi: 10.1038/ki.2015.88 25786101

[6] Pratschke J Wilhelm MJ Kusaka M Beato F Milford EL Hancock WW . Accelerated rejection of renal allografts from brain-dead donors. Ann Surg (2000) 232(2):263–71. doi: 10.1097/00000658-200008000-00017 PMC1421139 10903606

[7] Zitur LJ Chlebeck PJ Odorico SK Danobeitia JS Zens TJ Van Kooten C . Brain death enhances activation of the innate immune system and leads to reduced renal metabolic gene expression. Transplantation (2019) 3(9):1821–33. doi: 10.1097/TP.0000000000002744 PMC6713605 30964836

[8] Locke JE Segev DL Warren DS Dominici F Simpkins CE Montgomery RA . Outcomes of kidneys from donors after cardiac death: implications for allocation and preservation. Am J Transplant (2007) 7(7):1797–807. doi: 10.1111/j.1600-6143.2007.01852.x 17524076

[9] Barlow AD Metcalfe MS Johari Y Elwell R Veitch PS Nicholson ML . Case-matched comparison of long-term results of non-heart beating and heart-beating donor renal transplants. Br J Surg (2009) 96(6):685–91. doi: 10.1002/bjs.6607 19434702

[10] Solez K Colvin RB Racusen LC Haas M Sis B Mengel M . Banff 07 classification of renal allograft pathology: updates and future directions. Am J Transplant (2008) 8(4):753–60. doi: 10.1111/j.1600-6143.2008.02159.x 18294345

[11] Roberts MB Fishman JA . Immunosuppressive agents and infectious risk in transplantation: Managing the "net state of immunosuppression. Clin Infect Dis (2021) 73(7):e1302–17. doi: 10.1093/cid/ciaa1189 PMC8561260 32803228

[12] Betjes MGH Sablik KS Otten HG Roelen DL Claas FH de Weerd A . Pretransplant donor-specific anti-HLA antibodies and the risk for rejection-related graft failure of kidney allografts. J Transplant (2020) 2020:5694670. doi: 10.1155/2020/5694670 32099669 PMC7008278

[13] Dunn TB Noreen H Gillingham K Maurer D Ozturk OG Pruett TL . Revisiting traditional risk factors for rejection and graft loss after kidney transplantation. Am J Transplant (2011) 11(10):2132–43. doi: 10.1111/j.1600-6143.2011.03640.x PMC3184338 21812918

[14] Alfaro R Martínez-Banaclocha H Llorente S Jimenez-Coll V Galián JA Botella C . Computational prediction of biomarkers, pathways, and new target drugs in the pathogenesis of immune-based diseases regarding kidney transplantation rejection. Front Immunol (2021) 12:800968. doi: 10.3389/fimmu.2021.800968 34975915 PMC8714745

[15] Spolverato G Maqsood H Kim Y . Neutrophil-lymphocyte and platelet-lymphocyte ratio in patients after resection for hepato-pancreatico-biliary malignancies. J Surg Oncol (2015) 111(7):868–74. doi: 10.1002/jso.23900 25865111

[16] Naranjo M Agrawal A Goyal A Margonis G Luo T Ejaz A . Neutrophil-to-Lymphocyte ratio and platelet-to-Lymphocyte ratio predict acute cellular rejection in the kidney allograft. Ann Transplant (2018) 23:467–74. doi: 10.12659/AOT.909251 PMC6248021 29987271

[17] Vartolomei MD Kimura S Ferro M Vartolomei L Foerster B Abufaraj M . Is neutrophil-to-lymphocytes ratio a clinical relevant preoperative biomarker in upper tract urothelial carcinoma? a meta-analysis of 4385 patients. World J Urol (2018) 36(7):1019–29. doi: 10.1007/s00345-018-2235-5 29468284

[18] Kim ED Famure O Li Y Kim SJ . Uric acid and the risk of graft failure in kidney transplant recipients: a re-assessment. Am J Transplant (2015) 15(2):482–8. doi: 10.1111/ajt.13000 25612498

[19] Dahle DO Jenssen T Holdaas H Leivestad T Vårdal M Mjøen G . Uric acid has a J-shaped association with cardiovascular and all-cause mortality in kidney transplant recipients. Clin Transplant (2014) 28(1):134–40. doi: 10.1111/ctr.12290 24372653

[20] Kim DG Choi HY Kim HY Lee EJ Huh KH Kim MS . Association between post-transplant serum uric acid levels and kidney transplantation outcomes. PloS One (2018) 13(12):e0209156. doi: 10.1371/journal.pone.0209156 30550582 PMC6294369

[21] Isakov O Patibandla BK Shwartz D Mor E Christopher KB Hod T . Can uric acid blood levels in renal transplant recipients predict allograft outcome? Ren Fail (2021) 43(1):1240–9. doi: 10.1080/0886022X.2021.1969246 PMC8405090 34433378

[22] Khosla UM Zharikov S Finch JL Nakagawa T Roncal C Mu W . Hyperuricemia induces endothelial dysfunction. Kidney Int (2005) 67(5):1739–42. doi: 10.1111/j.1523-1755.2005.00273.x 15840020

[23] Penack O Peczynski C van der Werf S Finke J Ganser A Schoemans H . Association of uric acid levels before start of conditioning with mortality after allogeneic hematopoietic stem cell transplantation - a prospective, non-interventional study of the EBMT transplant complication working party. Haematologica (2020) 15(7):1977–83. doi: 10.3324/haematol.2019.228668 PMC7327652 31601686

