# Supplementary material for: Serum uric acid as a risk factor for rejection after deceased donor kidney transplantation: A mono-institutional analysis of paired kidneys
Source: Front Immunol. 2022 Dec 12;13:973425. doi: 10.3389/fimmu.2022.973425 (PMC9791182; doi:10.3389/fimmu.2022.973425)
Supplement: Supplementary file 1 [file Table_1.doc]

Supplementary table 1. Reference of blood and biochemical tests in authors’ institution.

| **Items** | **Reference** |
| --- | --- |
| Red blood cell count (10^12/L) | 3.8-5.1 |
| Hemoglobin (g/L) | 115-150 |
| Platelet count (10^9/L) | 100-300 |
| [White blood cell count (10^9/L)](../../../../D:/Download/%25E6%259C%2589%25E9%2581%2593%25E8%25AF%258D%25E5%2585%25B8/Dict/9.0.6.0/resultui/html/index.html#\\javascript:;) | 3.5-9.5 |
| ANC (10^9/L) | 1.8-6.3 |
| Total bilirubin (μmoI/L) | 5.0-28.0 |
| Alanine aminotransferase (IU/L) | < 40 |
| Aspartate aminotransferase (IU/L) | < 35 |
| Albumin (g/L) | 40.0-55.0 |
| Globulin (g/L) | 20.0-40.0 |
| Urea nitrogen (mmol/L) | 2.6-7.5 |
| Creatinine (μmoI/L) | 48-79 |
| Cystatin C (mg/L) | 0.51-1.09 |
| FBG (mmoI/L) | 3.9-5.9 |
| UA (μmoI/L) | 160-380 |
| ALC (×10^9/L) | 1.1-3.2 |
| AMC (×10^9/L) | 0.1-0.6 |
| HDL (mmoI/L) | > 1.04 |
| LDL (mmoI/L) | < 3.12 |
| Triglyceride (mmoI/L) | 0.29-1.83 |
| Cholesterol (mmoI/L) | 2.8-5.7 |
