# Supplementary figures and images for: Serum uric acid as a risk factor for rejection after deceased donor kidney transplantation: A mono-institutional analysis of paired kidneys
Source: Front Immunol. 2022 Dec 12;13:973425. doi: 10.3389/fimmu.2022.973425 (PMC9791182; doi:10.3389/fimmu.2022.973425)

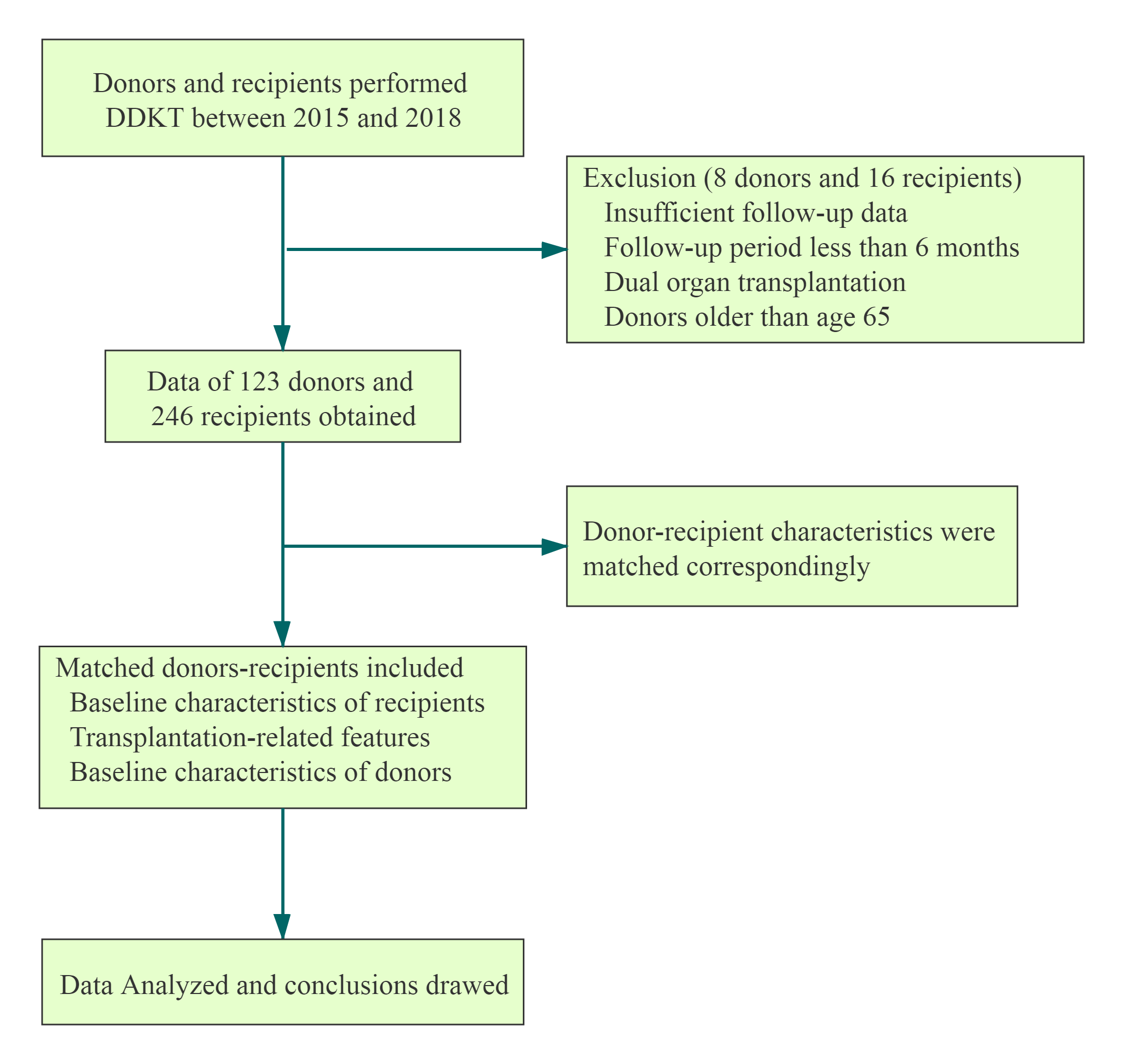

Supplement: Supplementary Figure 1 — Flow chart. [file Image_1.tif]
